# Herpes Simplex Virus Infection in Neonates and Young Infants with Sepsis

**DOI:** 10.5812/ircmj.14310

**Published:** 2014-02-05

**Authors:** Saeid Amel Jamehdar, Gholamali Mammouri, Mohammad Reza Sharifi Hoseini, Hosein Nomani, Monavvar Afzalaghaee, Hassan Boskabadi, Mohammad Hassan Aelami

**Affiliations:** 1 Department of Microbiology and Virology, Imam Reza Hospital, School of Medicine, Mashhad University of Medical Sciences, Mashhad, IR Iran; 2 Department of Pediatrics, Ghaem Hospital, School of Medicine, Mashhad University of Medical Sciences, Mashhad, IR Iran; 3 Department of Biostatistics, Mashhad University of Medical Sciences, Mashhad, IR Iran; 4 Department of Pediatrics and Infection Control & Hand Hygiene Research Center, Imam Reza Hospital, School of Medicine, Mashhad University of Medical Sciences, Mashhad, IR Iran

**Keywords:** Herpes Simplex, Infants, Polymerase Chain Reaction, Iran

## Abstract

**Background:**

Neonatal herpes infection is the most serious complication of Herpes Simplex Virus (HSV) infection during pregnancy and perinatal period. Few studies have reported neonatal HSV infection in developing countries.

**Objectives:**

The aim of this study was to detect the HSV infection among neonates and infants with sepsis.

**Materials and Methods:**

In a cross sectional study all infants aged less than 3 months, admitted to neonatal intensive care unit and pediatric emergency ward of Ghaem Hospital (a university hospital with 900 beds) in Mashhad (Northeast of Iran) with clinical diagnosis of sepsis and at least one inclusion criteria during one year from November 2009 to October 2010, were enrolled in the study. Polymerase chain reaction (PCR) was done on clinical samples obtained from patients.

**Results:**

Among 150 neonates and infants younger than 3 months old with sepsis, the PCR results for detecting the HSV DNA, were positive in 6 samples of 5 patients (3.3 %). None of the mothers had symptomatic HSV infection during delivery. The mean age of the patients was 18 days. Two of them died due to shock and disseminated intravascular coagulation (DIC).

**Conclusions:**

In neonates and infants with primary diagnosis of sepsis, HSV infection should be considered especially if the clinical condition does not improve after 48 hours of antibiotic therapy, and sepsis still exists with elevated liver enzymes.

## 1. Background

Herpes simplex virus infection can cause significant morbidity and mortality in neonates and infants. HSV transmission can occur in one of these three periods:, intrauterine, perinatal or postnatal, with estimated incidence rates of 5 %, 85 % and 10 %, respectively (1). The incidence of neonatal HSV infections in different parts of the world is 1.7–3/100000 live births in the UK (2), 9.6 cases/100000 births in the USA (3) and 3.2 cases per 100000 live births per year in the Netherlands (4). Although there are some studies about the prevalence of HSV type 2 in pregnant women in developing countries (5-9), but very few studies have reported the neonatal HSV infection in such countries (10, 11). Clinical manifestations in the first three weeks of life are localized skin/eye/mouth disease, encephalitis or disseminated disease. One third of HSV infected neonates lead to encephalitis and one fourth develop disseminated disease (12, 13). HSV infection is rarely considered, and antiviral therapy is advised only if there is a maternal history of HSV infection or if the child has suggestive ulcerative lesions.

## 2. Objectives

The aim of this study was to identify HSV infection among high risk neonates and infants with sepsis, which was considered as inclusion criteria in our center.

## 3. Materials and Methods

In a cross sectional study all infants younger than 3 months, who were admitted to the neonatal intensive care unit and pediatric emergency ward of Ghaem Hospital (a university hospital with 900 beds) in Mashhad (located in Northeast of Iran) with clinical diagnosis of sepsis and at least one of the inclusion criteria, were enrolled in this study (14-17) (Table 1). 

**Table 1.** The Inclusion Criteria

1	Vesicular Lesions of Skin and Mucus Membranes Without any Explanations
**2**	Repeated seizures (more than one time) without any explanations
**3**	If the clinical picture did not improve after 48 hours of antibiotic therapy
**4**	Sepsis with elevated level of liver enzymes
**5**	All ill and toxic neonates and infants are with shock, DIC^a^, decreased level of consciousness, apnea, metabolic acidosis and hypotonia
**6**	Mononuclear pleocytosis of CSF^a^ fluid
**7**	Positive mother history of genital herpes
**8**	Pneumonia with unknown cause
**9**	All neonates and infants who died during hospitalization
**10**	All neonates and infants with suspected TORCH^a^ infections

^a^ Abbreviations: CSF: cereberospinal fluid; DIC: disseminated intravascular coagulation; TORCH: indicates congenital infections including toxoplasma, rubella, cytomegalovirus, herpes simplex virus, etc.

The study period was from November 2009 to October 2010. Formal consents were obtained from the parents, the physicians in the neonatal and emergency wards collected demographic and clinical data including age (in days), sex, birth weight (kg), and gestational age (GA) (preterm: GA below 37 weeks; term: 37-week GA or more), mode of delivery [vaginal delivery vs. cesarean section (C/S)] , antibiotic use, fever (a rectal temperature higher than or equal to 38.0°C), hypothermia (a rectal temperature less than or equal to 36.4°C), respiratory insufficiency, skin lesions, neurological symptoms (seizures, hypotonia, and feeding problems), hepatosplenomegaly and DIC. In addition, genital HSV infection of the mother was recorded. Sampling was carried out by trained nurses. All laboratory tests were done by the central laboratory as part of routine lab tests. The laboratory results recorded were as follows: leukopenia (leukocyte count < 4.0 × 10^9^/l), leukocytosis (leukocyte count > 15.0×10^9^/l), thrombocytopenia (platelet count < 150×10^9^/l), C-reactive protein (CRP) > 6 mg/L, aspartate aminotransferase (AST) ≥ 60 U/L, alanine aminotransferase (ALT) > 30 U/L and, prothrombin time (PT) > 16, partial thromboplastin time (PTT) > 55 and blood PH < 7.2. Cereberospinal fluid (CSF) pleocytosis was defined as the presence of ≥ 25 white blood cells /mm^3^.

All the clinical samples from the patients including blood, CSF, skin and conjunctival swabs (in the case of skin vesicles) were sent to the Virology Department of Ghaem Hospital, Mashhad, Iran for PCR assay.

### 3.1. DNA Extraction

Viral genomic DNA was extracted from the samples using Invisorb Spin Virus DNA Mini kit protocol (Invitek GmbH, Germany) according to the manufacturer’s instructions. DNA samples were stored at -70 ºC prior to molecular diagnosis of HSV infection.

### 3.2. Viral Detection

**Figure 1.**
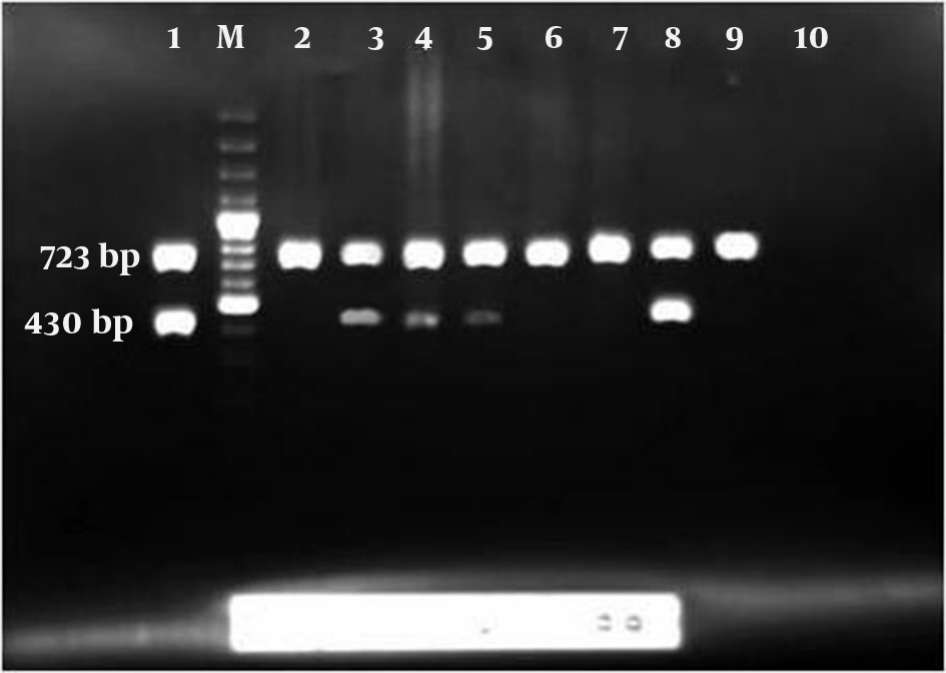
Result of HSV PCR No. 1 is positive control with 723 bp and internal control 430 bp, No. M is 100 bp DNA ladder, No. 2, 6, 7, 9 are negative samples, No. 3, 4, 5 and 8 are positive samples, No. 10 is a negative control.

HSV diagnosis was carried out by HSV PCR amplification kit (Amplisens, Russia). After DNA amplification, data analysis was done based on the presence or absence of specific bands of amplified DNA by electrophoresis on an agarose gel (1.5 %). (Figure 1) 

In order to typing the HSV, amplification of an HSV gene fragment was carried out using Herpes Simplex Virus Type 1 and 2 kits, as instructed by the manufacturer (GenID, GmbH, Germany). Briefly, a fragment of highly conserved region of the HSV genome is amplified together with a fragment of human glyceraldehydes- 3- phosphate dehyrogenase (GAP-DH) gene, as an extraction and amplification control, in a multiplex PCR using a mixture of biotin labeled primers. Characterization of PCR fragments and differentiation of HSV types are subsequently carried out by reverse hybridization assay (18). The research protocol was approved by Mashhad University of Medicine Institutional Review Board; code No. 89283-12/11/2010. The results were analyzed using SPSS software version 11.5 (Illinois, USA) and the data were presented as medians with interquartile ranges.

## 4. Results

In one year period from November 2009 to October 2010, 150 neonates and infants with sepsis and at least one inclusion criteria, were enrolled in our study. There were 85 boys and 61 girls with mean age of 12.95 ± 1.4 days and 16.8 ± 1.82 days, respectively. The PCR for DNA of HSV were positive in six samples obtained from five patients (3.3 %) (Table 2). All belonged to HSV type 2. There was a patient from nursery with unknown birth history. None of the mothers of the patients with known birth history had symptomatic HSV infection during delivery. The mean age of the patients was 18 days, interquartile range was 3 - 45 days (Table 2). 

**Table 2.** Demographic Features of the Patients ^a^

Patient	Sex	Age	Mode of delivery	Preterm	Clinical picture	Laboratory findings	Site of positive HSV PCR	Outcome
**1**	Male	3 days	C/S	Yes	Sepsis with Shock	Prolonged PT, Thrombocytopenia, Indirect Hyperbilirubinemia, metabolic acidosis	Blood	Death
**2**	Male	28 days	C/S	Yes	Cholestasis and DIC	Leukocytosis, Thrombocytopenia, Prolonged PT & PTT, Direct Hyperbilirubinemia	Blood	Death
**3**	Female	45 days	Normal	No	Hydrocephaly and fever	Indirect Hyperbilirubinemia, CSF pleocytosis with, RBCs in CSF, Normal CSF sugar, elevated CSF protein	Blood & CSF	Alive
**4**	Male	6 days	Unknown	No	Culture negative Sepsis, Aseptic meningitis	Prolonged PT, CSF pleocytosis, RBCs in CSF, Normal CSF sugar, elevated CSF protein	CSF	Alive
**5**	Male	8 days	C/S	No	Culture negative Sepsis	Thrombocytopenia, Positive CRP	Blood	Alive

^a^Abbreviations: BCs: red blood cells; C/S: cesarean section; CRP: C-reactive protein; CSF: cerebrospinal fluid; DIC: disseminated intravascular coagulation; HSV: Herpes Simplex Virus; PCR: polymerase chain reaction; PT: prothrombin time; PTT: partial thromboplastin time.

Two of the patients had history of preterm labor and three other had birth weight less than 2500 g. Among five patients with HSV infection showed some clinical manifestations including, poor feeding in 3 (60 %), jaundice in 3 (60 %), fever in 2 (40 %), apnea in 2 (40 %), cyanosis in 2 (40 %), bulging of anterior fontanel in 1 (20 %), respiratory distress in 1 (20 %) and conjunctivitis in 1 (20 %) and no skin lesion was detected. Thrombocytopenia in 3 (60 %), indirect hyperbilirobinemia in 2 (40 %), cholestasis in 1 (20 %), prolonged PT and PTT in 1 (20 %), elevated liver transaminases in 1 (20 %) and metabolic acidosis in 1 (20 %) were the most important laboratory findings. Chest X-rays of two patients were normal. None of these patients received acyclovir. Two died due to shock and DIC.

## 5. Discussion

This is the first article about the rate of herpes simplex virus among high risk neonates and infants with sepsis as characterized by inclusion criteria. This led to find high proportion of HSV infection (3.3 %) among septic neonates compare to low prevalence of HSV in similar studies. In a study by Caviness et al. the prevalence of HSV infection was 0.2 % in hospitalized neonates admitted to a pediatric emergency department. In this study, the prevalence of HSV infection (0.2 %) was not statistically different from that of bacterial meningitis (0.4 %) but was lower than that of all serious bacterial infections (4.6 %) in 5817 hospitalized neonates admitted during a 5-year period (19). In a retrospective cohort study by Ambroggio et al. 460 patients < 60 days of age with a primary discharge diagnosis of HSV were studied. Intravenous acyclovir was administered and they were discharged between January 1, 2003 and December 31, 2005. Of these patients, 21 (5 %) died (20). In a study by Kropp et al. fifty-eight cases of neonatal herpes simplex virus were detected (5.9 cases per 100000 live births) (21). The majority of neonatal HSV infections are acquired during delivery, although in utero and postnatal infections occur (17). In sub-Saharan Africa, high HSV2 rates (30 - 80 %) observed in women. In South America, the prevalence of HSV2 ranges from 20 to 40 %. Lower values (10 - 30 %) have been seen in the general population in Asian countries (22). In a study in Iran, the prevalence of HSV type 2 among pregnant women was 8.25 % (5).

Neonatal infections caused by peripartum HSV transmission are classified into 3 groups including disseminated HSV infection causing severe multiorgan dysfunction and has a high mortality rate if left untreated; Herpesviral encephalitis, which causes neurologic morbidity among most of the survivors; and HSV infection localized to the skin, eye, and/or mouth, may progress to encephalitis or disseminated disease if left untreated (23). The clinical outcome of two neonates in our study was in consistent with disseminated disease; a preterm neonate with septic shock and the other with cholestasis and DIC. The final outcome was poor for both of them.

In a study by Kropp et al. localized HSV infection was reported in 59.6 % of the patients and disseminated disease and central nervous system disease were reported in 17.5 % and 22.8 % of the cases, respectively (21). In the present study, two of five infants with HSV infection were preterm and died. In a study by O’Riordan et al. the relative risk for death of preterm infants with HSV infection was3.7 times more than term infants with HSV infection. In this study, all infants with disseminated disease (n = 9) died, whereas the three infants with encephalitis survived. In agreement with our study, herpes simplex virus infections in preterm infants usually presented during the first 2 weeks of life and had a high incidence of dissemination (24). Kimberlin et al. noted that elevation of AST level to >10 times of the normal level at the initiation of acyclovir, DIC, coma, pneumonitis, HSV 1 infection and delayed antiviral treatment have all been associated with increased mortality rate (25). In our study, none of the mothers had symptomatic HSV infection during delivery. In a study by O’Riordan et al. no mother had herpes simplex virus lesions at delivery, but a history of genital herpes simplex or other sexually transmitted infections was prevalent among the mothers (24). In Long et al. study, only 11 % of the mothers had history of genital HSV infection (16).

In another study by Kropp et al. 40 % of the mothers had no history of genital herpes infection before delivery, and intrapartum genital lesions were present in only in 1 of 58 cases (21) The mean age of our patients was 18 days. In a study about the incidence of neonatal HSV infection across the United States in 2006, the median age at the time of admission was 10 days and 25 % of the admissions were on the first day of birth (3). Ambroggio et al. found that in 460 patients younger than 60 days of age with a primary discharge diagnosis of HSV infection, the median age was 16 days (interquartile range: 8 – 31 days) (20) Long et al. reported that age of ≤ 21 days at onset of symptoms comprised 90% of all infected infants with HSV admitted to a children's hospital in North Philadelphia (16). No skin lesions were observed in our patients. In a study by Long et al. it was shown that 75 % of HSV infected infants had central nervous system (CNS) infection, including 40 % of those who had mucocutaneous lesions. Cultures of mucocutaneous lesions for HSV detection were positive in eight of ten patients (16). Poor feeding in 3 (60 %), jaundice in 3 (60 %), fever in 2 (40 %), apnea in 2 (40 %), cyanosis in 2 (40 %), bulging of anterior fontanel in 1 (20 %) and respiratory distress in 1 (20 %), were some of clinical outcomes of the patients in the present study. These signs might be caused by other diseases such as hyaline membrane disease, intraventricular hemorrhage, necrotizing enterocolitis, and various ocular or cutaneous illnesses; infections with group B *Streptococcus*, *Staphylococcus aureus*, *Listeria monocytogenes*, and Gram-negative bacteria, which can be misdiagnosed with neonatal HSV infection. Infection with varicella-zoster virus, enteroviruses, and cytomegalovirus could have clinical outcomes similar to the neonatal HSV infection, Toxoplasmosis and rubella infection. In a study by Fidler et al. eight cases of confirmed disseminated HSV infection were identified. An important factor for delayed treatment seems to be lack of awareness of this disease amongst the clinicians (17).

With the advent of polymerase chain reaction (PCR) assay, a sensitive and specific diagnosis of HSV infections can be made from blood and/or CSF within 24 hours (26). The American Academy of Pediatrics Committee on Infectious Diseases recommends: HSV infection should be considered in “neonates with fever, irritability, and abnormal CSF findings” especially in the presence of convulsions (27). HSV infection should be considered in differential diagnosis of sepsis even though in mothers without any history of HSV infection. In babies with outcomes similar to the HSV infection, proper laboratory specimens should be taken and intravenous acyclovir administration (60 mg/kg/day) should be started immediately (17). In our study all the infectious agents were HSV type 2. Genital infection is typically caused by HSV type 2 although the ratio of infections caused by HSV type 1 is increasing (28, 29). HSV-1 as a genital infection has a higher risk of transmission to the neonate than HSV-2 (30). Three of four cases with known birth history had history of C/S. For decreasing the incidence of neonatal HSV infections, cost-effective routine screenings to detect the maternal HSV antibodies are controversial (31-33). Also, the C/S reduces, the HSV transmission from mother to child and may increase the maternal morbidity and mortality, compared with vaginal deliveries thus, are only recommended in the presence of documented primary HSV infection or visible genital lesions at the time of delivery (34). Since most physicians in emergency departments and neonatal wards did not consider HSV detection in the differential diagnosis of sepsis, we proposed to consider HSV in high risk infants with sepsis especially in patients diagnosed with sepsis and negative sepsis culture and infants with shock and DIC without any maternal history of HSV infection. After taking laboratory specimens, appropriate antiviral drug should be immediately administered for these patients.

## References

[1] Kimberlin DW (2007). Herpes simplex virus infections of the newborn.. Semin Perinatol..

[2] Tookey P, Peckham CS (1996). Neonatal herpes simplex virus infection in the British Isles.. Paediatr Perinat Epidemiol..

[3] Flagg EW, Weinstock H (2011). Incidence of neonatal herpes simplex virus infections in the United States, 2006.. Pediatrics..

[4] Poeran J, Wildschut H, Gaytant M, Galama J, Steegers E, van der Meijden W (2008). The incidence of neonatal herpes in The Netherlands.. J Clin Virol..

[5] Ziyaeyan M, Japoni A, Roostaee MH, Salehi S, Soleimanjahi H (2007). A serological survey of Herpes Simplex Virus type 1 and 2 immunity in pregnant women at labor stage in Tehran, Iran.. Pak J Biol Sci..

[6] Chen XS, Yin YP, Chen LP, Yu YH, Wei WH, Thuy NT (2007). Herpes simplex virus 2 infection in women attending an antenatal clinic in Fuzhou, China.. Sex Transm Infect..

[7] Biswas D, Borkakoty B, Mahanta J, Walia K, Saikia L, Akoijam BS (2011). Seroprevalence and risk factors of herpes simplex virus type-2 infection among pregnant women in Northeast India.. BMC Infect Dis..

[8] Dolar N, Serdaroglu S, Yilmaz G, Ergin S (2006). Seroprevalence of herpes simplex virus type 1 and type 2 in Turkey.. J Eur Acad Dermatol Venereol..

[9] Kirakoya-Samadoulougou F, Nagot N, Defer MC, Yaro S, Fao P, Ilboudo F (2011). Epidemiology of herpes simplex virus type 2 infection in rural and urban Burkina Faso.. Sex Transm Dis..

[10] Yaghobi R, Zamani S, Gramizadeh B, Rahsaz M (2010). Etiology of DNA virus infections in liver transplant recipients with neonatal hepatitis.. Transplant Proc..

[11] Gupta A, Rani PK, Bagga B, Dore P, Mittal A, Jalali S (2010). Bilateral herpes simplex-2 acute retinal necrosis with encephalitis in premature twins.. J AAPOS..

[12] Whitley R, Arvin A, Prober C, Corey L, Burchett S, Plotkin S (1991). Predictors of morbidity and mortality in neonates with herpes simplex virus infections. The National Institute of Allergy and Infectious Diseases Collaborative Antiviral Study Group.. N Engl J Med..

[13] Whitley R, Arvin A, Prober C, Burchett S, Corey L, Powell D (1991). A controlled trial comparing vidarabine with acyclovir in neonatal herpes simplex virus infection. Infectious Diseases Collaborative Antiviral Study Group.. N Engl J Med..

[14] Rudnick CM, Hoekzema GS (2002). Neonatal herpes simplex virus infections.. Am Fam Physician..

[15] Malm G (2009). Neonatal herpes simplex virus infection.. Semin Fetal Neonatal Med..

[16] Long SS, Pool TE, Vodzak J, Daskalaki I, Gould JM (2011). Herpes simplex virus infection in young infants during 2 decades of empiric acyclovir therapy.. Pediatr Infect Dis J..

[17] Fidler KJ, Pierce CM, Cubitt WD, Novelli V, Peters MJ (2004). Could neonatal disseminated herpes simplex virus infections be treated earlier?. J Infect..

[18] Ziyaeyan M, Alborzi A, Borhani Haghighi A, Jamalidoust M, Moeini M, Pourabbas B (2011). Diagnosis and quantitative detection of HSV DNA in samples from patients with suspected herpes simplex encephalitis.. Braz J Infect Dis..

[19] Caviness AC, Demmler GJ, Almendarez Y, Selwyn BJ (2008). The prevalence of neonatal herpes simplex virus infection compared with serious bacterial illness in hospitalized neonates.. J Pediatr..

[20] Ambroggio L, Lorch SA, Mohamad Z, Mossey J, Shah SS (2009). Congenital anomalies and resource utilization in neonates infected with herpes simplex virus.. Sex Transm Dis..

[21] Kropp RY, Wong T, Cormier L, Ringrose A, Burton S, Embree JE (2006). Neonatal herpes simplex virus infections in Canada: results of a 3-year national prospective study.. Pediatrics..

[22] Weiss H (2004). Epidemiology of herpes simplex virus type 2 infection in the developing world.. Herpes..

[23] Arvin AM, Whitley RJ, Gutierrez KM, Remington JS, Klein JO, Wilson CB, Baker CJ (2006). Herpes simplex virus infections.. Infectious Diseases of the Fetus and Newborn Infant..

[24] O'Riordan DP, Golden W, Aucott SW (2006). Herpes simplex virus infections in preterm infants.. Pediatrics..

[25] Kimberlin DW, Lin CY, Jacobs RF, Powell DA, Frenkel LM, Gruber WC (2001). Natural history of neonatal herpes simplex virus infections in the acyclovir era.. Pediatrics..

[26] Malm G, Forsgren M (1999). Neonatal herpes simplex virus infections: HSV DNA in cerebrospinal fluid and serum.. Arch Dis Child Fetal Neonatal Ed..

[27] (2009). Red Book: 2009 Report of the Committee on Infectious Diseases..

[28] Vyse AJ, Gay NJ, Slomka MJ, Gopal R, Gibbs T, Morgan-Capner P (2000). The burden of infection with HSV-1 and HSV-2 in England and Wales: implications for the changing epidemiology of genital herpes.. Sex Transm Infect..

[29] Drake S, Taylor S, Brown D, Pillay D (2000). Improving the care of patients with genital herpes.. BMJ..

[30] Brown EL, Gardella C, Malm G, Prober CG, Forsgren M, Krantz EM (2007). Effect of maternal herpes simplex virus (HSV) serostatus and HSV type on risk of neonatal herpes.. Acta Obstet Gynecol Scand..

[31] Rouse DJ, Stringer JS (2000). An appraisal of screening for maternal type-specific herpes simplex virus antibodies to prevent neonatal herpes.. Am J Obstet Gynecol..

[32] Thung SF, Grobman WA (2005). The cost-effectiveness of routine antenatal screening for maternal herpes simplex virus-1 and -2 antibodies.. Am J Obstet Gynecol..

[33] Baker D, Brown Z, Hollier LM, Wendel GJ, Hulme L, Griffiths DA (2004). Cost-effectiveness of herpes simplex virus type 2 serologic testing and antiviral therapy in pregnancy.. Am J Obstet Gynecol..

[34] (2000). ACOG practice bulletin. Management of herpes in pregnancy. Number 8 October 1999. Clinical management guidelines for obstetrician-gynecologists.. Int J Gynaecol Obstet..

